# Preoperatıve Ultrasound-Guıded Suprascapular Nerve Block for Postthoracotomy Shoulder Paın^☆^

**DOI:** 10.1016/j.curtheres.2012.12.004

**Published:** 2013-06

**Authors:** Emine Özyuvaci, Onat Akyol, Tolga Şitilci, Türkan Dübüs¸, Hakan Topac¸ogˇlu, Hülya Leblebici, Alican Ac¸ikgöz

**Affiliations:** 1 Department of Anesthesiology and Pain Medicine, İstanbul Training and Research Hospital, İstanbul, Turkey; 2 Department of Thoracic Surgery, İstanbul Training and Research Hospital, İstanbul, Turkey; 3 Department of Emergency Medicine, İstanbul Training and Research Hospital, İstanbul, Turkey

**Keywords:** postthoracotomy, shoulder pain, suprascapular nerve, ultrasound

## Abstract

**Background:**

Acute postthoracotomy pain is a well-known potential problem, with pulmonary complications, ineffective respiratory rehabilitation, and delayed mobilization in the initial postoperative period, and it is followed by chronic pain. The type of thoracotomy, intercostal nerve damage, muscle retraction, costal fractures, pleural irritation, and incision scar are the most responsible mechanisms.

**Objective:**

Our aim was to assess whether preoperative ultrasound suprascapular nerve block with thoracic epidural analgesia was effective for postthoracotomy shoulder pain relief.

**Methods:**

Thirty-six American Society of Anesthesiologist classification physical status I–III patients (2011–2012), with a diagnosis of lung cancer and scheduled for elective open-lung surgery, were prospectively included in the study. Eighteen of the patients received an ultrasound-guided suprascapular nerve block with 10-mL 0.5% levobupivacaine, using a 22-gauge spinal needle, 1 hour before operation (group S); 18 other patients had thoracic epidural analgesia only, and no nerve block was performed. Standard general anesthesia was administered. Degree of shoulder pain was assessed by a blinded observer when discharging patients from the recovery room, and thereafter at 1, 3, 6, 12, 24, 36, 48, and 72 hours on infusion at rest and 12, 24, 36, 48, and 72 hours on coughing. The same blinded observer also recorded the total amount of epidural levobupivacaine and fentanyl used by the 2 groups.

**Results:**

In the suprascapular block group, the total amount of levobupivacaine (*P =* 0.0001) and fentanyl (*P* = 0.005) used postoperatively was statistically lower than in the epidural group. Visual analogue scale measurements in the suprascapular group were statistically significantly lower at 0, 1, 3, 6, 12, 24, 36, and 48 hours than those in the epidural group, both at rest and coughing.

**Conclusion:**

Postthoracotomy shoulder pain reduces patient function and postsurgical rehabilitation potential after thoracotomy, and various studies on explaining the etiology and management of postthoracotomy shoulder pain have been conducted. Theories of the etiology involved either musculoskeletal origin or referred pain. In this study, we concluded that preoperative ultrasound-guided suprascapular nerve block with thoracic epidural analgesia could achieve effective shoulder pain relief for 72 hours postoperatively, both at rest and coughing.

## Introduction

Acute postthoracotomy pain is a well-known potential problem, with pulmonary complications, ineffective respiratory rehabilitation, and delayed mobilization in the initial postoperative period, and it is followed by chronic pain. The type of thoracotomy, intercostal nerve damage, muscle retraction, costal fractures, pleural irritation, and incision scar are the most responsible mechanisms.^1^

Long-term postthoracotomy pain is a sequela problem after thoracotomies. Katz et al.^2^ reported a retrospective study in which 52% of patients reported chronic pain, suggesting that early postoperative pain was a significant reason for pain becoming chronic; they used aggressive pain management for acute postthoracotomy pain. Obata et al^3^ concluded, in a randomized, prospective trial, that intraoperative and postoperative thoracic epidural analgesia decreased chronic pain significantly compared with only postoperative epidural analgesia after thoracotomy. In contrast to this finding, Kavanagah et al.^4^ concluded that preemptive analgesia was ineffective for chronic thoracotomy pain.

Ipsilateral shoulder pain also coincides with the postoperative period and has been reported in >75% of various studies. A possible stimulus that might explain this referred pain is that the pleural sides of the mediastinum and diaphragm are conducted via the phrenic nerve. Other reported etiologies are transection of the major bronchus, patient position during surgery, straining of muscles and joints around the shoulder, and thoracotomy tube.^5^ Different analgesic techniques, both pharmacological and interventional, are being used by anesthesiologists for postoperative pain relief. Burgess et al^6^ reported that in 19 of 45 patients with moderate to severe shoulder pain after pulmonary resection, both IV opioids and thoracic epidural analgesia limited effectivity. The reported managements for shoulder pain were blockage of the interscalene brachial plexus, superficial cervical plexus, suprascapular nerve, phrenic nerve, and stellate ganglion. Intraoperative local anesthetic injection and NSAIDs were also additive as multimodal treatments.^6^ Ng et al^7^ described 5 cases of ipsilateral postthoracotomy shoulder pain in which they performed an interscalene brachial plexus block and treated the pain effectively with thoracic epidural analgesia.

The suprascapular nerve originates from the brachial plexus and has many sensorial afferents from most of the shoulder joint. In a detailed survey of the international literature, we were unable to find any studies on the effectiveness of preoperative ultrasound-guided suprascapular nerve block with thoracic epidural analgesia for severe postthoracotomy ipsilateral shoulder pain. Therefore, we conducted this prospective, randomized study to assess the hypothesis of whether blockade of the suprascapular nerve, by directly visualizing this nerve preoperatively with ultrasound, could reduce the severity of ipsilateral shoulder pain.

## Materials and Methods

Ethical approval for this study was provided by the Institutional Review Board of Istanbul Education and Research Hospital in January 2011. Written informed consent was obtained from each patient before inclusion in the study. Thirty-six American Society of Anesthesiologist classification physical status I–III patients, with a diagnosis of lung cancer and scheduled for elective open-lung surgery between 2011 and 2012 were prospectively included in the study. The same surgeons performed a lobectomy through a posterolateral thoracotomy incision in the sixth intercostal space. Patients with severe cardiopulmonary or renal disease, allergies to local anesthetics, known psychiatric or neurologic diseases, alcohol or narcotics abuse, contraindications to thoracic epidural or suprascapular block, preexisting shoulder problems, or previous thoracotomy history were excluded.

After the patients arrived in the operating theater, a 16-gauge IV cannula was inserted into the forearm opposite to the surgical side, and then standard premedication was given with 2-mg midazolam IV A 20-gauge epidural catheter was placed in the T6–T7 interspace using the midline approach and a saline loss of resistance technique under a C-arm scope, through an 18-gauge Tuohy epidural needle. The catheter was advanced 3 to 5 cm inside the epidural space, and a test dose of 3-mL lidocaine 2% with epinephrine 1:200,000 was given to exclude misplacement of the catheter. Eighteen of the patients received an ultrasound-guided suprascapular nerve block with 10-mL 0.5% levobupivacaine using a 22-gauge spinal needle 1 hour before the operation (group S), and the other 18 patients received thoracic epidural analgesia only, and no nerve block was performed. An epidural block was induced with 10-mL 0.5% levobupivacaine + 2.5 μg/mL fentanyl at anesthesia induction, and then maintained with 5 mL/h infusion and 10-mL bolus per 45 minutes during surgery. All patients were monitored with 2-lead electrocardiography (leads II and V_5_) for heart rate and ST-segment changes, with noninvasive pulse oximetry (SpO_2_), and an arterial catheter for invasive blood pressure monitoring and blood gas analysis. Airway pressures, ventilation parameters, inspired oxygen concentration, expired end-tidal carbon dioxide concentration (EtCO_2_), and end-tidal sevoflurane concentration were also monitored.

Standard general anesthesia was induced with propofol (2.5 mg/kg), fentanyl (1 μg/kg), and vecuronium (0.1 mg/kg). A double-lumen endobronchial tube was then placed, and the lungs ventilated using a 50% oxygen in air mixture with a Drager–Julian pressure-cycled ventilator (Drager, Lubeck, Germany). Patients were then placed in the lateral decubitus position, and neuromuscular block was maintained with IV boluses of 0.01 mg/kg vecuronium per hour.

General anesthesia was maintained with 2% end-tidal concentration sevoflurane and 40 to 45 EtCO_2_ concentration; pressure-controlled mandatory ventilation mode was used. Intraoperative arterial pressure and heart rate were maintained within 20% of baseline values by altering the concentration of sevoflurane, or by administering IV fluids and ephedrine boluses. At the end of the operation, at the start of skin closure, sevoflurane inhalation was discontinued, and the residual neuromuscular block was antagonized (neostigmine 2 mg and atropine 1 mg IV). When the patient was judged to be awake, extubation was performed with adequate oxygenation (SpO_2_ 90% at room air). All patients had only 1 chest drain tube. The patient was then transferred to the intensive care unit. Postoperative analgesia consisted of a patient-controlled epidural analgesia infusion of 0.5% levobupivacaine + 2.5 μg/mL fentanyl (baseline infusion: 5 mL/h; incremental dose: 8 mL; lockout period: 20 min). Postoperatively, patients were assessed and their data recorded by a blinded observer. The degrees of pain at rest (visual analogue scale [VAS] R) and on coughing (VAS C) were assessed using a 10-cm VAS, with zero signifying no pain and 10 signifying the worst pain. Degree of shoulder pain was assessed by an observer when discharging patients from the recovery room and then at 1, 3, 6, 12, 24, 36, 48, and 72 hours on infusion at rest and 12, 24, 36, 48, and 72 hours on coughing. The same observer also recorded the total amount of epidural levobupivacaine and fentanyl consumption by the groups.

## Results

In this prospective, double-blind study, all statistical analysis was performed with the Number Cruncher Statistical System (Kaysville, Utah) statistical software program. Data are expressed as mean (SD). Statistical comparisons were made by Student’s *t*-test or the Mann–Whitney *U* test for continuous variables and χ^2^ test or Fisher’s exact test for categorical variables. A *P* value < 0.05 was considered statistically significant.

There were no statistically significant differences between the groups in terms of demographics (Table I), American Society of Anesthesiologist classification distribution (Table II), or patient weight (Table III). In the suprascapular block group, total amounts of postoperative levobupivacaine (*P =* 0.0001) and fentanyl (*P =* 0.005) were statistically lower than in the epidural group (Table III).

**Figure 1:**
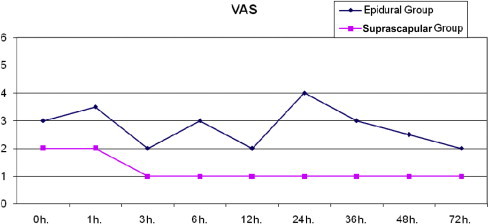
Visual analogue scale (VAS) at rest.

**Figure 2:**
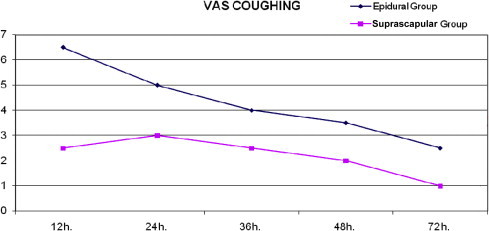
Visaul analogue scale (VAS) at coughing.

VAS measurements at rest in the suprascapular group were statistically significantly lower at 0, 1, 3, 6, 12, 24, 36, and 48 hours than those in the epidural group (Table IV and Figure 1). There were no statistically significant differences between the epidural and suprascapular groups in VAS measurements (*P =* 0.479) at 72 hours. VAS coughing measurements in the suprascapular group were statistically significantly lower at 12, 24, 36, 48, and 72 hours than those in the epidural group (Table V and Figure 2).

## Discussion

Postthoracotomy shoulder pain reduces patient function and postsurgical rehabilitation potential after thoracotomy. The incidence of postthoracotomy shoulder pain in various studies ranges from 21% to 97%, and thoracic epidural analgesia is ineffective for eliminating this pain after surgery. General approaches to postthoracotomy shoulder pain consist of regular administration of NSAIDs and acetaminophen, intraoperative injection of local anesthetic into the pericardiac fat pad, interscalene brachial plexus block, stellate ganglion blockade, and direct blockade of the phrenic nerve.^8^

Various studies were conducted to explain the etiology and management of postthoracotomy shoulder pain. Theories on the etiology involved either musculoskeletal origin or referred pain.

In a 2008 review about shoulder pain, Gerner^1^ stated that the pain was relatively resistant to intravenous opioids and was only partially relieved by NSAIDs. Related mechanisms were transection of a major bronchus, ligamentous strain from malposition or surgical mobilization of the scapula, pleural irritation due to the thoracotomy tube, or referred pain from irritation of the pericardium or mediastinal and diaphragmatic pleural surfaces.^1^ Scawn et al^9^ reported in prospective, randomized, placebo-controlled studies that phrenic nerve block had a significant role in the etiology of transmitting postthoracotomy shoulder pain, and that its blockade was found to be an effective treatment in the management of this pain. Barak et al,^10^ in another double-blind study, concluded that an interscalene brachial plexus block could be a beneficial treatment for postthoracotomy shoulder pain. Garner and Coats^11^ reported on a patient who experienced a significant reduction in postthoracotomy shoulder pain after ipsilateral stellate ganglion block.

We designed a prospective, randomized study in which suprascapular nerve block with ultrasound guidance was effective for postthoracotomy shoulder pain. However, many etiologies and theories were considered for explaining ipsilateral postthoracotomy shoulder pain; the theory most emphasized in the literature was that irritation of the pericardium or mediastinal and diaphragmatic pleural surfaces resulted in pain transferred to the ipsilateral shoulder via the phrenic nerve.

In contrast, Mark and Brodsky^5^ mentioned shoulder position and mechanical traction of the thoracic muscular and skeletal structures for shoulder pain mechanism. In a 2006 review, Gottschalk et al^12^ stated that chronic pain after thoracic surgery could be localized or radicular in nature, and that the pain could be exacerbated by movement of the ipsilateral shoulder. Considering these theories, suprascapular nerve block seems to have a partial role in pain conduction from the shoulder.

The suprascapular nerve has both motor and sensory components originating from the ventral rami of C4, C5, and C6 spinal nerves and goes through the upper trunk of the brachial plexus. The nerve runs inferiorly, laterally, and posteriorly to enter the supraspinatus fossa via the suprascapular notch and innervates the supraspinatus and infraspinatus muscles. The suprascapular nerve innervates 70% of the shoulder joint and supplies the posterior glenohumeral joint capsule, acromioclavicular joint, subacromial bursa, and coracoclavicular ligament.^13^ Suprascapular nerve blockade has been effective in treating chronic shoulder pain and improving joint motion.^14^

Chan et al^15^ showed in their study that ultrasound could determine the size, depth, and exact location of the brachial plexus and its neighboring structures. Real-time ultrasound imaging could help guide the block needle to reach target nerves with fewer attempts.^15^ Siegenthaler et al^16^ reported that visualization of the suprascapular nerve with ultrasound was better in the supraclavicular region compared with the supraspinous fossa for effectiveness of both nerve block and pain relief. Williams et al^17^ compared the ultrasound-guided technique with nerve stimulation and found that ultrasound guidance was superior in nerve blocking. Using a high-resolution ultrasonography technique, anesthetists could visualize nerve structures directly for nerve blocks at all levels and monitor the distribution of local anesthetics. Saha et al^18^ performed a large-sized retrospective study and stated that physical examination of the shoulder before applying a suprascapular nerve block was beneficial in reducing ipsilateral shoulder pain. Their decision was that if the pain was of musculoskeletal origin, then a suprascapular nerve block could be carried out postoperatively.^18^

We would like to point out a possible limitation of this study, in that we did not perform a physical examination of whether the shoulder joint experienced tenderness before we administered the nerve block.

Tan et al^19^ suggested that suprascapular nerve block was ineffective in the management of postthoracotomy shoulder pain. They postulated that pain from distraction of the shoulder capsule was unlikely to be a major component of shoulder pain, and based on Mackenzie’s convergence theory, the somatic afferent suprascapular nerve to spinal cord is amplified by visceral pain coming from the diaphragm via the phrenic nerve.^19^ They concluded that the phrenic nerve is a major target for postthoracotomy shoulder pain. In contrast to our ultrasound-guided nerve block at the supraspinous fossa, Tan et al^19^ applied a suprascapular nerve block by using a nerve stimulator in the form of a needle 1 cm above the midpoint of the scapular spine. Similar to this study, Martinez-Barenys^20^ et al reported that ipsilateral periphrenic fat pad infiltration was superior to a block of the ipsilateral suprascapular nerve in reducing the incidence and severity of postthoracotomy shoulder pain. However, they determined that a limitation of their study was a possible lack of precision of the block, which resulted from performing the suprascapular nerve block without using electromyography or ultrasound guidance, which are more effective than blind injection into the suprascapular fossa.

Peng et al^21^ presented a case report about ultrasound-guided suprascapular nerve block and confirmed the findings with anatomic cadaveric dissection, concluding that by positioning the ultrasound probe in the coronal plane over the suprascapular fossa with a slight anterior tilt, the nerve can be visualized on the ﬂoor of the scapular spine between the scapular notch and the spinoglenoid notch. In a prospective study, Beach et al^22^ found that positive motor response to nerve stimulation did not increase the success rate of the nerve block when a satisfactory image of the suprascapular nerve was not obtained. In addition, false-negative rates proved that ultrasound-guided blocks were usually effective, even in the absence of a motor response.^22^

As a result of this study, we concluded that preoperative ultrasound-guided suprascapular nerve block with thoracic epidural analgesia could be part of a multimodal analgesic treatment for postthoracotomy shoulder pain management.

## Conflict of Interests

The authors have indicated that they have no conflicts of interest regarding the content of this article.

## Figures and Tables

**Table I:** Demographic characteristics.

Characteristics	Epidural Group	Suprascapular Group	Values
Age, mean (SD)	57.5 (8.21)	61.83 (8.69)	*t* = 1.53; *P* = 0.133
Gender			
Male, n (%)	14 (77.80)	13 (72.20)	χ² = 0.15
Female, n (%)	4 (22.20)	5 (27.80)	*P* = 0.700

**Table II:** American Society of Anesthesiologist classification (ASA).

ASA	Epidural Group, n (%)	Suprascapular Group, n (%)
I	4 (22,20)	4 (22.20)
II	11 (61.10)	12 (66.70)
III	3 (16.70)	2 (11.10)

*χ*^2^ = 0.24.*P* = 0.885

**Table III:** Weight and total levobupivacaine (levo) + fentanyl (fenta) consumption.

Characteristics	Epidural Group, mean (SD)	Suprascapular Group, mean (SD)	*t*	*P*
Weight, kg	64.39 (6.71)	63.33 (6.34)	0.48	0.486
Total levo, mg/kg	1.45 (0.28	1 (0.22)	5.42	0.0001
Total fenta, μg/kg	5.36 (0.95	4.1± (1.3)	3.31	0.002

**Table IV:** Postoperative visual analogue scale (VAS) at rest.

VAS	Epidural Group	Suprascapular Group	MW	*P*
O h				
Mean (SD)	2.72 (0.75)	1.67 (1.28)	93	0.021
Median (IQR)	3 (2–3)	2 (0–3)		
1 h				
Mean (SD)	3.72 (1.87)	1.72 (0.9)	37.5	0.0001
Median (IQR)	3.5 (2.75–4)	2 (1–2)		
3 h				
Mean (SD)	2.78 (1.11)	1.72 (1.18)	77	0.005
Median (IQR)	2 (2–3.25)	1 (1–3)		
6 h				
Mean (SD)	3.06 (1.21)	1.5 (0.99)	49.5	0.0001
Median (IQR)	3 (2–4)	1 (1–2)		
12 h				
Mean (SD)	2.94 (1.26)	1.78 (1.26)	72.5	0.003
Median (IQR)	2 (2–4)	1 (1–2.25)		
24 h				
Mean (SD)	4.06 (1.51)	1.44 (0.62)	20.5	0.0001
Median (IQR)	4 (3–5.25)	1 (1–2)		
36 h				
Mean (SD)	2.72 (1.13)	1.44 (0.62)	49	0.0001
Median (IQR)	3 (2–3.25)	1 (1–2)		
48 h				
Mean (SD)	2.61 (1.42)	1.22 (0.55)	52.5	0.0001
Median (IQR)	2.5 (2–3.25)	1 (1–1)		
72 h				
Mean (SD)	1.67 (1.72)	1.11 (0.32)	141	0.479
Median (IQR)	2 (0–3)	1 (1–1)		
Fr	34.41	10.54		
*P*	0.0001	0.229		

Fr, frequency; IQR, interquartile range; MW, mean and weighted average.

**Table V:** Postoperative visual analogue scale (VAS) at coughing.

VAS Coughing	Epidural Group	Suprascapular Group	MW	*P*
12 h				
Mean (SD)	6.94 (1.39)	2.94 (1.39)	11	0.0001
Median (IQR)	6.5 (6––8)	2.5 (2–4)		
24 h				
Mean (SD)	4.89 (1.18)	2.67 (0.84)	17	0.0001
Median (IQR)	5 (4–6)	3 (2–3)		
36 h				
Mean (SD)	3.89 (1.18)	2.5 (0.99)	63	0.001
Median (IQR)	4 (3–5)	2.5 (2–3)		
48 h				
Mean (SD)	3.56 (1.34)	2.33 (1.14)	81	0.009
Median (IQR)	3.5 (2–4.25)	2 (1–3)		
72 h				
Mean (SD)	2.5 (1.43)	1.5 (0.86)	85.5	0.012
Median (IQR)	2.5 (2–4)	1 (1–2)		
Fr	51.01	20.18		
*P*	0.0001	0.0001		

Fr, frequency; IQR, interquartile range; MW, mean and weighted average.
